# Assessment of tribological performance and thermal stability of metakaolin-based geopolymer composites reinforced with high TiO_2_ concentration

**DOI:** 10.1038/s41598-026-54064-4

**Published:** 2026-05-27

**Authors:** Mohamed Ali Hassan, Shiamaa Awys, Mahmoud Abd El-Aleem Ali Ali EL-Remaily

**Affiliations:** 1 Mechanical Department, Faculty of Technology and Education, Sohag University, Sohag, Egypt; 2 Department of Chemistry, Faculty of Science, Sohag University, Sohag, 82524 Egypt; 3 Faculty of Science, Sohag National University, Sohag, 82524 Egypt

**Keywords:** Metakaolin-based geopolymer, Titanium dioxide (TiO_2_), Thermal stability, DSC, TGA, Wear resistance, Coefficient of friction (COF)

## Abstract

This study investigates the influence of titanium dioxide (TiO_2_) incorporation (0–50 wt.%) on the structural, thermal, and tribological properties of metakaolin-based geopolymer composites (GPCs). The bulk density of the composites increased progressively from 1.81 g/cm^3^ for the control sample to 2.86 g/cm^3^ at 50 wt.% TiO_2_, while apparent porosity decreased from 33.47 to 23.48%. Water absorption was correspondingly reduced from 8.43 to 5.34% after 24 h immersion, confirming the pore-filling effect of TiO_2_. XRD and FTIR analyses indicated the coexistence of amorphous aluminosilicate gel, residual quartz, and anatase reflections, with Ti–O and Ti–O–Si vibrations confirming the physical embedding of TiO_2_ without disrupting the geopolymeric framework. SEM micrographs revealed that higher TiO_2_ content led to a denser morphology with fewer pores, confirming densification. DSC/TGA revealed that TiO_2_ addition enhanced stability and reduced low-temperature mass loss. Pin-on-disc testing showed that adding 40 wt.% TiO_2_ significantly improved tribological performance, reducing the wear rate from 3.45 × 10^−5^ to 1.12 × 10^−5^ mm^3^/N m and the steady-state friction coefficient from 0.36 to 0.29. These results confirm the dual role of TiO_2_ as a microstructural densifier and a reinforcing agent, enabling the development of geopolymer composites with enhanced durability, thermal stability, and wear resistance suitable for high-performance structural applications in extreme environments.

## Introduction

Bricks and cement remain fundamental to the construction industry^1^. However, their significant environmental footprint and energy-intensive production have catalyzed the search for sustainable alternatives^2–4^. Geopolymer composite materials (GCM) have emerged as a promising solution, recognized for their exceptional sustainability and mechanical robustness^5,6^. GCM synthesis, which alkali-activates aluminosilicate precursors like metakaolin or fly ash, reduces emissions by 80–90% compared to traditional Portland cement^7–9^. GCMs are highly valued for their superior properties, including high mechanical strength^10^, chemical resistance^11^, and, notably, exceptional thermal stability at temperatures exceeding^12,13^. Recent technological advancements have explored enhancing these matrices by incorporating various fillers to tailor their performance for specialized industrial applications^14–16^. While many studies focus on lightweight foams for insulation^17,18^, there is a critical need for high-density, wear-resistant, and thermally stable geopolymers capable of withstanding harsh mechanical and thermal environments^19,20^. GCM represents a vital component of both current and future sustainable cementitious binder systems^8^. They can be synthesized from a diverse array of precursors, all rich in silica and alumina, which vary significantly in their global accessibility, reactivity, and crucially, cost-effectiveness^21,22^. These materials were characterized using a cubic lattice of interconnected SiO_4_ and AlO_4_ tetrahedra in a three-dimensional network^13^. Fly ash (FA) and metakaolin (MK), two alternate sources of silica and alumina, are reacted with strong alkaline solutions to create GCM^23^. Potassium hydroxide (KOH), sodium hydroxide (NaOH), and soluble silicates such as sodium silicate are commonly found in these solutions^24^. In this reaction, silicon dioxide (SiO_2_) and dissolved aluminum oxide (Al_2_O_3_) species go through a process known as geopolymerization^25,26^. This reaction forms an amorphous, three-dimensional aluminosilicate network^27^. The creation of lightweight geopolymer composites has advanced significantly in recent years^17^. Researchers have explored adding various fillers to enhance mechanical, thermal, acoustic, and moisture-absorption properties, thereby broadening application potential across industries^28^.

Natural and synthetic fibers, industrial by-products (like metakaolin, fly ash, and blast furnace slag)^29^, lightweight aggregates (like perlite and vermiculite)^18^, and recycled materials (like crushed glass and construction waste)^30^ are just a few of the fillers that can be used in the production of geopolymers. These additives significantly alter the microstructure and characteristics of geopolymer foams, affecting behaviors like thermal conductivity, linear shrinkage, mechanical strength, and rheology^28^. Kaolinite and other silicon-and aluminum-rich clays have a crystalline structure that is ideal for the development of geopolymers^31^. The layered clay mineral kaolinite has a lamellar structure with tetrahedra connected to octahedral alumina by oxygen atoms^32^. Kaolinite is extremely reactive due to its unique structure and glassy texture. In strongly alkaline environments, its high silicon and aluminum oxide content fosters a three-dimensional, branching molecular network^33^. Metakaolin is widely used in the manufacturing of geopolymers for many reasons. Initially, it is a common industrial mineral with uniform qualities^34^. Metakaolin is a valuable admixture due to its high porosity, large surface area, high absorption capacity, and ability to form strong coordination bonds upon activation^35^. The early-stage performance and reaction kinetics of alkali-activated metakaolin geopolymers are dependent on curing conditions^36,37^, dosage, and concentration of raw materials^38,39^. Finally, metakaolin production has a significantly lower environmental impact than the production of many industrial fillers^40^. Researchers have recently incorporated metal oxide into geopolymers to improve their performance and durability. Titanium dioxide (TiO_2_) enhances cementitious systems by improving durability and microstructure^41,42^.

Previous studies by Guzmán-Aponte et al.^43^ and Duan et al.^44^ have shown that it can promote a denser microstructure and reduce micro-cracking. However, most existing literature focuses on low replacement levels (up to 10%). There is a significant scientific gap regarding the behavior of geopolymer composites at high loading levels, where the filler may transition from a simple additive to a primary structural component.

This study explores the combined physical, mechanical, and tribological properties of metakaolin-based geopolymers reinforced with high concentrations (10–50 wt.%) of microparticles, moving beyond traditional insulation applications. This study investigates the impact of high filler loading on the densification, wear mechanisms, and thermal stability of GCMs. These composites exhibit promising abrasion resistance and structural reliability in thermal environments, making them suitable for demanding industrial applications, as evidenced by their microstructure and mechanical integrity.

## Materials and methods

### Material

El-Nasr Mining Company of Egypt supplied the kaolin (Al_2_Si_2_O_5_(OH)_4_) with a size of < 75 μm used to produce metakaolin (MK), which is rich in alumina and silica. The process of calcination took place at 800°C in an electric furnace for 180 min to obtain MK and increase its reactivity for geopolymerization^45,46^. The MK was sieved to a particle size of 35 μm, and its chemical composition was determined by X-ray fluorescence (XRF) analysis, as shown in Table 1.

**Table 1:** Chemical composition of MK as investigated by XRF.

SiO_2_	Al_2_O_3_	Na_2_O	K_2_O	TiO_2_	Fe_2_O_3_	SO_3_	CaO	MgO	LOI (loss on ignition)
50.61	46.29	0.5	0.5	0.48	0.45	0.45	0.21	0.17	0.34

Titanium dioxide powder with an average size (< 30 μm) and a 99.5% purity supplied from EL-Goumhouria Chemical Company (Cairo, Egypt). The alkali activator, which is composed of sodium hydroxide (NaOH) and liquid sodium silicate (Na_2_SiO_3_), was purchased from the Silica Egypt Company in Alexandria, Egypt.

### Geopolymer synthesis

#### Alkaline activator (AA)

A solution of alkaline activator (AA) with a concentration of 8 mol/L was prepared by dissolving NaOH pellets with a purity of 99% in distilled water and stirring it for 15 min at room temperature. To fully activate the alkaline solution and initiate geopolymerization, the alkaline activator solution and liquid sodium silicate were mechanically stirred in a 1:2 volume ratio and aged for 48 h.

#### Geopolymer preparation

**Fig. 1:**
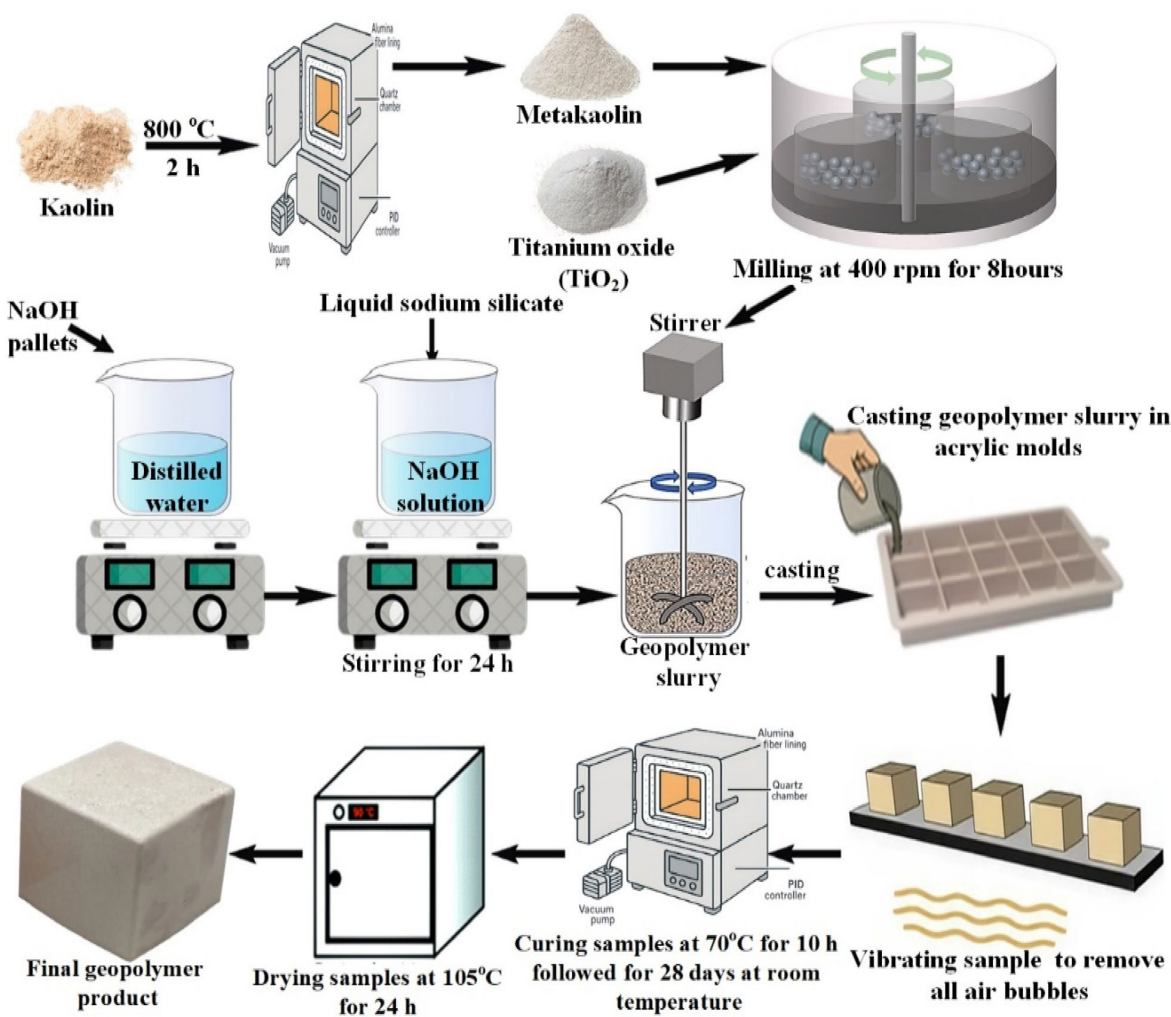
Synthesis steps of GPC samples.

Geopolymer composite powders with titanium dioxide content (x = 0, 10, 20, 30, 40, and 50 wt.%) were mechanically mixed with MK for 3 h to ensure uniform component distribution. An alkaline solution was then progressively added to the prepared mixtures and stirred with a mechanical mixer until a uniform mortar was formed. The mortars were cast as 50 × 50x50 mm^3^ cubes in acrylic molds. The mold is briefly vibrated to remove air bubbles and compact the paste. The molded specimens were first cured for 10 h at 70°C, and then they were allowed to cure at room temperature for 28 days. Finally, the cubes were dried in an oven set at 105°C for 24 h to prevent the geopolymerization reaction and ensure consistent moisture content for further characterization. Figure 1 displays a schematic diagram of the synthesis process steps of the GPC samples.

### Characterization and analysis

The apparent volume of permeable voids (AVPV) test on the 50 mm × 50 mm × 50 mm dimension cubical-shaped samples was determined in accordance with (ASTM C 642-97,2008) to evaluate the porosity variation among the various mixes^47^. The samples were initially maintained at 105°C for 24 h, then cooled to 25°C, and their masses (M_I_) were determined. Samples were boiled for 5 h in a container, then cooled to 25 °C, and their saturation mass (MS) was measured. Subsequently, samples suspended in water at 25°C were measured (Mf). AVPV was then estimated using the following formula:

1 $$\mathrm{A}\mathrm{V}\mathrm{P}\mathrm{V} \mathrm{\%}=\frac{{\mathrm{M}}_{\mathrm{s}}-{\mathrm{M}}_{\mathrm{I}}}{{\mathrm{M}}_{\mathrm{s}}-{\mathrm{M}}_{\mathrm{f}}} \times 100$$

Total porosity percentage (%) and bulk density of the produced GPC samples were investigated using an analytical balance according to ASTM C373-88,2006^48^. The water absorption test was calculated according to Neville’s method^49,50^. The sample’s weight was measured after 28 days of air curing, followed by 30 min in distilled water. After removing the samples from the water, the mass differences were promptly noted. After a further 24-h curing period in distilled water, masses were recorded to determine water absorption. The compressive strength of the various hardened pastes was investigated using an Instron 3369 universal test machine equipped with a 50 kN a deformation rate of 1 mm/min. For each parameter, five samples were tested to obtain an average compressive strength value. Samples were polished to provide parallel, flat surfaces for even compressive strength testing. The phase characteristics of both raw material powders and hardened GPC samples were determined by X-ray diffraction (XRD) type Bruker D8 using CuKα radiation (λ = 0.15406 nm). The scan rates were investigated with a 0.02° step size in a range from 5° to 90° for the raw materials powder pattern and 5° to 90° for the hardened pastes. To prepare the samples for microstructure inspection, the surface was ground and polished using 600, 800, 1000, and 1200 grit silicon carbide (SiC) papers. Microstructural characterization was performed using a Field Emission Scanning Electron Microscope (FE-SEM) (FE-SEM, ZEISS-Sigma 500 FE-SEM, Sohag, Egypt). Bruker Vertex FT-IR spectroscopy was used to identify functional groups formed after polymerization. The wavenumber of the FT-IR spectra ranged from 400 to 4000 cm^-1^. Approximately 2 mg of the paste was thoroughly ground with 20 times its weight of KBr, which was previously dried in an oven.

Thermal stability of the geopolymer pastes was assessed using thermogravimetric analysis (TGA) and differential scanning calorimetry (DSC) techniques. Both analyses were performed using Shimadzu instruments (TGA-50 and DSC-60, respectively) from Kyoto, Japan. To prevent thermal oxidation, the measurements were conducted under a nitrogen (N_2_) atmosphere with a heating rate of 10°C/min. The temperature range for the analysis extended from ambient conditions to 950°C.

Tribological behavior, including wear rate and coefficient of friction, was assessed utilizing an oscillating ball-on-disk tribometer in accordance with ASTM G 133–10^51^. The experimental conditions involved a 3 mm radius aluminum oxide ball as the counterface, an average sliding speed of 30 mm/s, a normal load of 5 N, and ambient conditions characterized by room temperature and 35–40% relative humidity. Wear volume loss was quantified by analyzing the wear track profile using a profilometer. Concurrently, the coefficient of friction was continuously monitored throughout the test duration using a force sensor.

## Results and discussion

### Phase composition of raw material and produced GPC

**Fig. 2:**
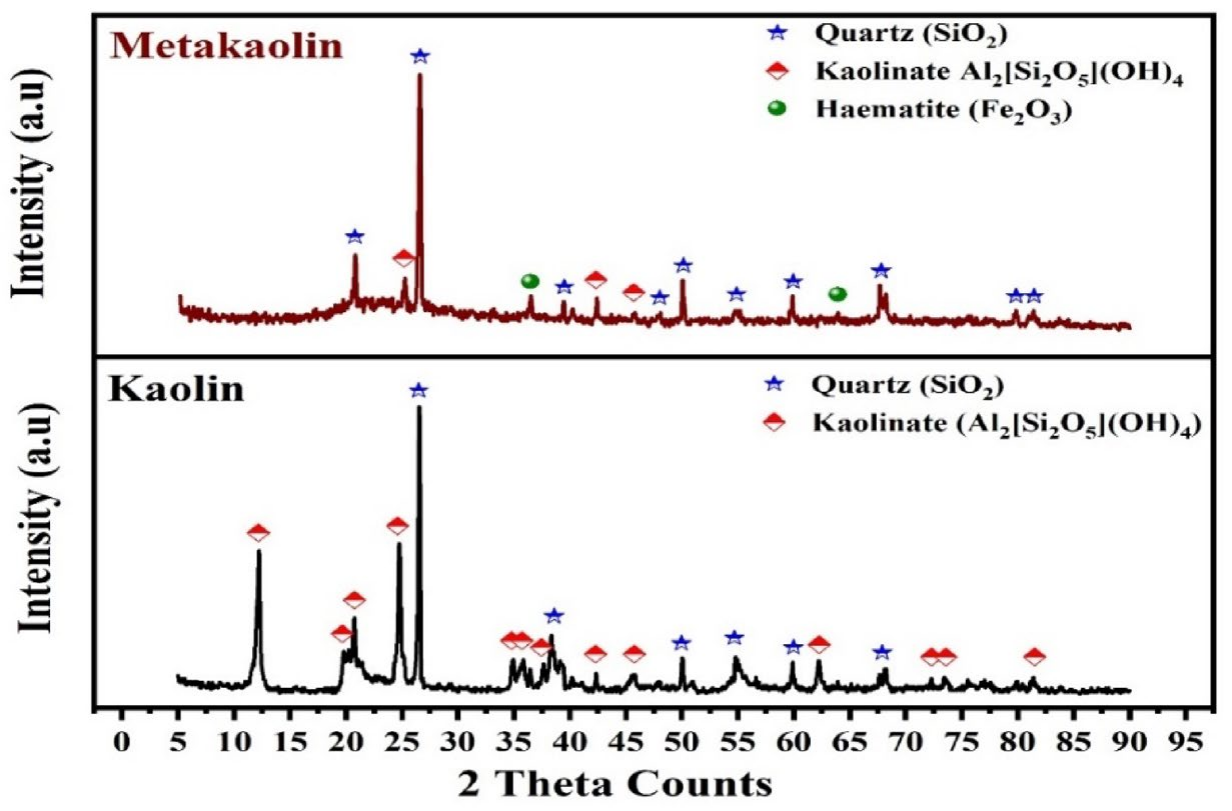
XRD patterns of the raw materials (kaolin and metakaolin).

Figure 2 shows the XRD patterns of the raw powder materials kaolin and metakaolin. The pattern of kaolin reveals that it is mainly composed of quartz (SiO_2_) (PDF#01–086–1628) and kaolinite (Al_2_[Si_2_O_5_] (OH)_4_) (PDF#96-900-9231). Also, the XRD pattern of metakaolin displays a typical broad reflection hump between 2θ (20° to 30°), which is dispersed to the amorphous phase^52^. Furthermore, quartz phases are detected in the pattern of metakaolin at 2θ = 26.7° and 21°. This result is attributed to the fact that at a calcination temperature of 800°C the crystalline phase content decreased and the crystal structure of kaolinite was eliminated with a transition from the crystalline to amorphous profile^53^. The other mineral phase, which appeared on the metakaolin pattern,was hematite (Fe_2_O_3_) (PDF # 04-003-2900). The hematite phase formed due to the dehydroxylation of goethite^23^.

**Fig. 3:**
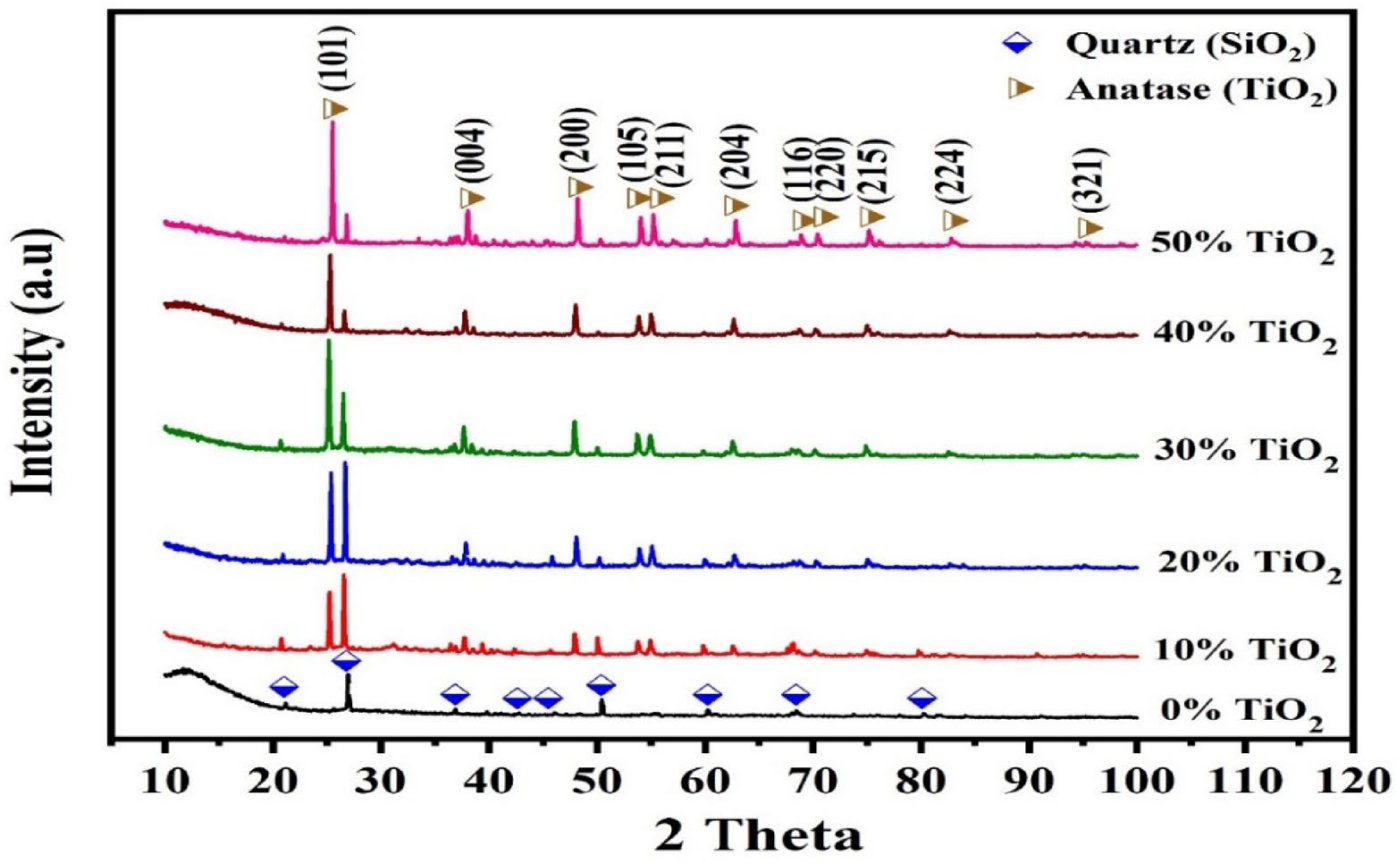
XRD patterns of the prepared GPC.

The diffractograms of the produced GPC with TiO_2_ addition are given in Fig. 3. This indicates the presence of the α quartz phase in all GPC samples, characterized by diffraction peaks at 2θ 21° and 26.75°^52^. The XRD broadening of the 0% TiO_2_ sample shows an asymmetric hump spanning the 2θ account range between 20° and 35°, which is representative of the predominantly amorphous aluminosilicate gel (N–A–S–H) that forms during geopolymerization. Furthermore, XRD analysis showed sharp reflection peaks at 2θ values of 20.86° and 26.7° attributable to α-quartz (SiO₂), which is attributed to a residual quartz in the kaolin/metakaolin precursor. This case of amorphous hump and α-quartz (SiO₂) is a fingerprint that was widely reported for kaolin-derived geopolymers^54^.

Upon the addition of TiO_2_, the diffractograms exhibit sharp, well-defined Bragg reflections at 2θ = 25.23°, 37.78°, 48.15°, 53.94°, 55.17°, and 62.73°, which perfectly align with the (101), (004), (200), (105), (211), and (204) planes of the anatase phase^55^. Notably, the absence of a reflection at 27.4° (2θ) indicates that no phase transformation from anatase to rutile occurred during the processing or curing stages^56^. As the TiO_2_​ loading increases from 10 to 50%, the intensity of the anatase reflections grows monotonically. This trend is associated with a progressive attenuation of the amorphous hump and quartz reflections, primarily resulting from the volumetric displacement of the geopolymer matrix by the crystalline filler. Also, this observation is attributed to a physical dilution effect and the high crystallinity of the titania filler rather than a chemical alteration of the geopolymer network^43,57^. Furthermore, the peak locations for quartz remain unchanged across all samples, specifically the reflection at 2θ 26.7°, which is consistent with the presence of unreacted α-quartz from the precursor^58^. The consistency of the peak positions and the absence of additional crystalline reflections (e.g., titanium silicates) indicate that TiO_2_ functions predominantly as a chemically inert reinforcing filler within the geopolymer matrix, attributed to behavior that aligns with recent observations on the phase stability of reinforced sustainable binders^7,16^. This stable integration is crucial for maintaining the structural integrity of the composite at high filler concentration.

### Microstructural evaluation

**Fig. 4:**
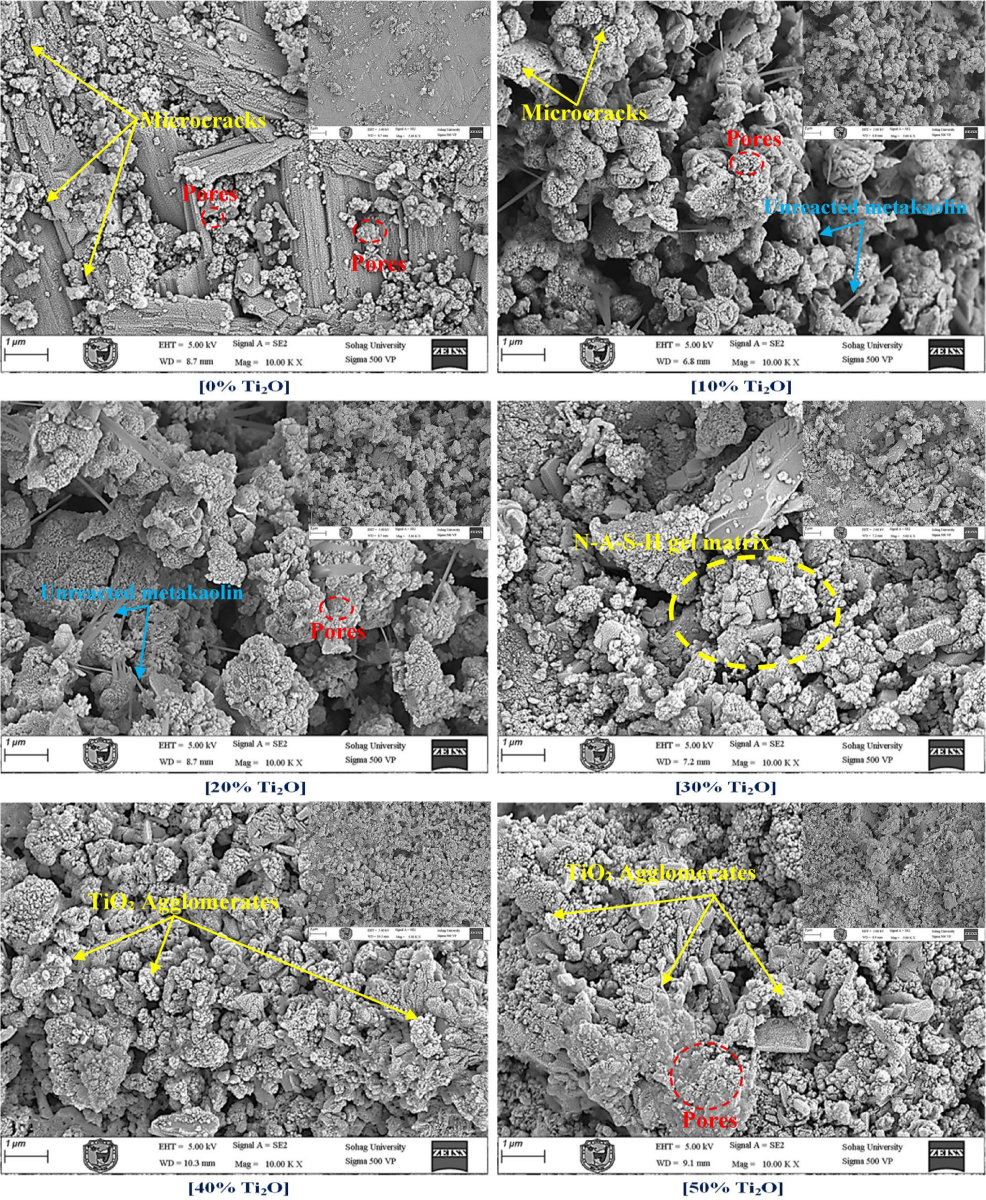
SEM micrographs of GPC samples with varying TiO_2_ concentration.

Figure 4 compiles representative FE-SEM micrographs of the GPC samples at various magnifications to elucidate the morphological evolution with loading. The control sample (0 wt.%) exhibits a typical geopolymeric morphology characterized by a dense, amorphous N-A-S–H gel with its characteristic globular "cauliflower-like" features^59^. A more detailed examination reveals a heterogeneous microstructure characterized by micro-cracks and residual capillary voids. These features coexist with laminar relics originating from partially reacted metakaolin and angular unreacted quartz grains^60^. With a 10–20 wt.% addition, a significant microstructural densification is observed. The micro-particles < 30μm effectively act as a physical filler, occupying the pre-existing voids and refining the pore structure. This pore-filling effect reduces the prevalence of open cavities, resulting in a more compact and continuous matrix, which is expected to enhance the mechanical integrity and wear resistance^56^ .At 30 wt% loading, while the matrix remains largely consolidated, the onset of particle agglomeration becomes evident at the inter-particle necks. As the concentration reaches 40 wt%, the micrographs reveal a transition toward a filler-dominated regime. This stage is characterized by the formation of prominent TiO_2_ agglomerates and the emergence of interfacial micro-voids at the boundaries between the crystalline aggregates and the geopolymer binder. This loading regime is marked by heightened agglomeration, which induces interfacial micro-porosity between the TiO_2​_ clusters and the binder, a phenomenon consistent with the microstructural defects and void formations reported in recent studies on high-filler cementitious systems^61–63^. Finally, at 50 wt.%, the microstructure is dominated by massive, consolidated aggregates forming a rough particle carpet. The high solids fraction leads to a dilution of the geopolymeric binder, partially disrupting the gel continuity and resulting in a coarser pore structure^64,65^. The retention of discrete, crystalline particles without secondary reaction products, as confirmed by the XRD results (Fig. 3), validates that it acts as a stable, inert reinforcement even at these exceptionally high loading levels.

### Fourier transform infrared spectroscopy (FTIR)

**Fig. 5:**
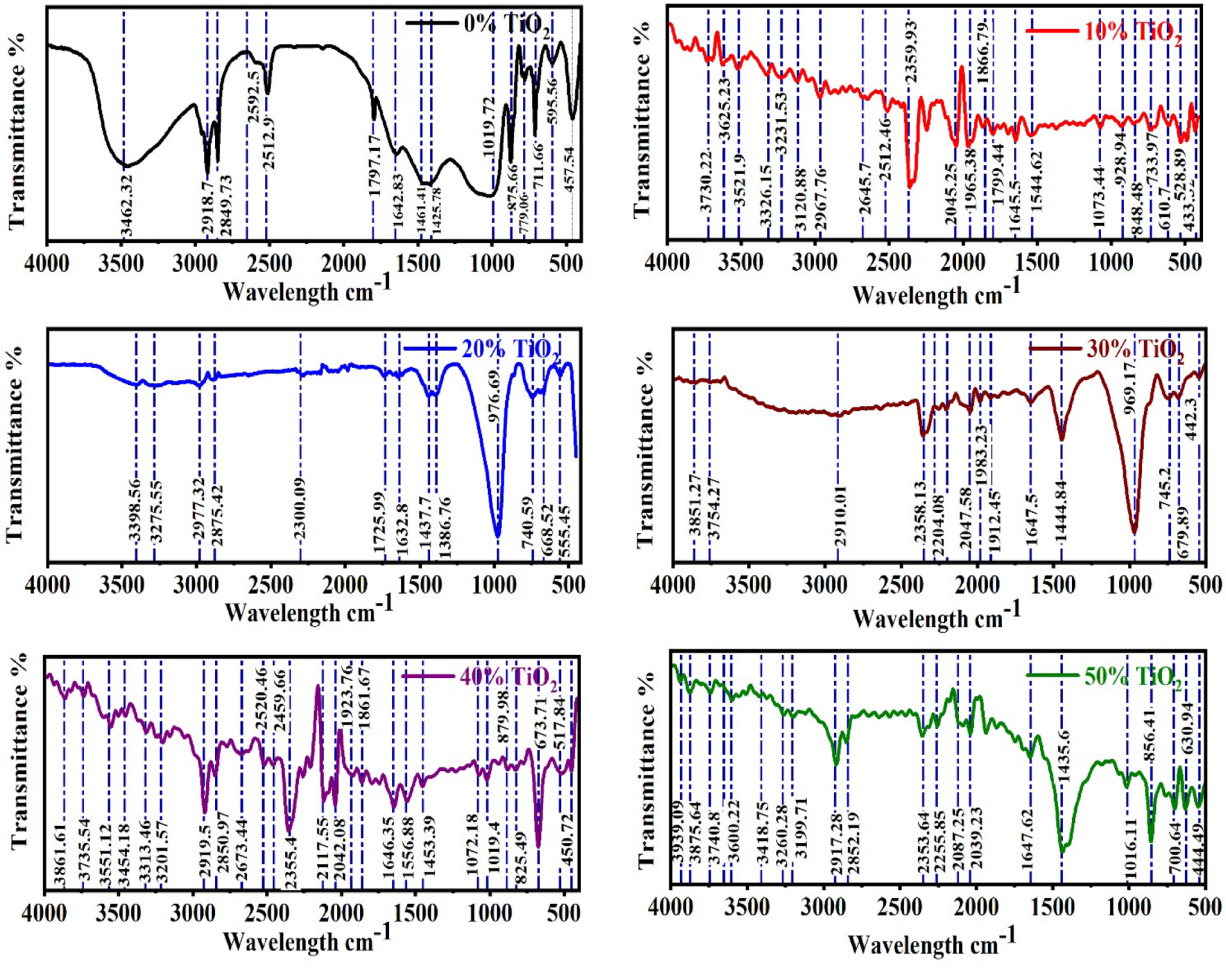
FTIR spectra of GPC samples with varying TiO_2_ concentration.

Figure 5 presents the FTIR spectra of the synthesized GPC containing varying proportions of TiO_2_. The spectra exhibit the canonical vibrational fingerprints of aluminosilicate frameworks, which undergo systematic modifications upon TiO_2_ incorporation. In the unmodified control sample (0 wt% TiO_2_), a broad absorption band centered at 3460 cm⁻^1^ is assigned to the O–H stretching vibrations of surface hydroxyl groups and physisorbed water molecules. Complementarily, the band at 1640 cm⁻^1^ corresponds to the H–O–H bending of interlayer or molecularly bound water within the geopolymeric network^66,67^. The main asymmetric stretching of Si–O–T (T = Si or Al) appears at 1019 cm⁻^1^, characteristic of the amorphous aluminosilicate gel phase^68^. With progressive TiO_2_ addition, this band exhibits slight wavenumber shifts and a reduction in intensity, implying that inert TiO_2_ particles partially disrupt the polymeric gel structure while not chemically integrating into the aluminosilicate framework^57^. Meanwhile, absorption features within the 450–750 cm⁻^1^ region are ascribed to Ti–O stretching and Ti–O–Si linkages. Their progressive intensification with increasing TiO_2_ loading provides spectroscopic evidence for the physical incorporation and persistence of anatase crystallites within the matrix, consistent with XRD observations. The sharp bands observed between 470 and 520 cm⁻^1^ further confirm the persistence of residual quartz, in agreement with diffraction data^69^. Moreover, TiO_2_-modified samples display attenuated intensities of the water-related O–H and H–O–H bands, suggesting enhanced microstructural densification and a reduction in free water retention. This interpretation is further corroborated by SEM evidence, where TiO_2_ appears to refine the pore architecture without initiating the formation of new crystalline aluminosilicate phases. Collectively, these spectral features affirm that TiO_2_ functions primarily as a physically dispersed filler, promoting pore refinement and densification rather than undergoing chemical incorporation into the geopolymeric gel^70^.

### Water absorption characteristics

Table 2 summarizes the bulk density, apparent porosity, and water absorption behavior of the synthesized GPC samples reinforced with varying TiO_2_ amounts from 0 up to 50 wt.%. The results unequivocally demonstrate the pronounced influence of TiO_2_ incorporation on the physical performance of the GPC samples. A systematic increase in bulk density is observed with progressive TiO_2_ addition, from 1.81 g/cm^3^ in the control blank sample to 2.855 g/cm^3^ at 50 wt.% TiO_2_. This densification effect can be primarily ascribed to the pore-filling capability of TiO_2_ particles, which efficiently occupy interstitial voids and capillary spaces, thereby reducing overall porosity and enhancing matrix compactness^71^. Correspondingly, the apparent porosity decreases significantly, from 33.47% at 0 wt.% TiO_2_ to 23.48% at 50 wt.% TiO_2_, confirming the densification of the matrix.

**Table 2:** Bulk density, apparent porosity, apparent volume of permeable voids (AVPV) values, and water absorption test.

Samples	Bulk density g/cm3	Apparent porosity (%)	AVPV (%)	Initial Mass (g)	Mass (g) after 30min	Increment %	Mass (g) after 24 h	Increment %
0 TiO_2_	1.81	33.47	15.84	226.35	238.62	5.42	245.43	8.43
10 TiO_2_	2.019	28.65	14.17	238.5	247.55	3.78	255.74	7.23
20 TiO_2_	2.228	26.24	13.57	252.74	260.68	3.14	269.83	6.76
30 TiO_2_	2.437	24.32	12.93	267.13	274.72	2.84	283.64	6.18
40 TiO_2_	2.284	23.7	12.09	284.32	291.17	2.41	300.84	5.81
50 TiO_2_	2.855	23.48	11.68	308.09	314.58	2.12	324.54	5.34

The inclusion of the apparent volume of permeable voids (AVPV) provides an additional parameter to assess the pore connectivity within the geopolymer matrix. As shown, the AVPV values decrease progressively from 15.84% at 0 wt.% TiO_2_ to 11.68% at 50 wt.% TiO_2_, confirming that TiO_2_ addition reduces the accessible void fraction and improves matrix compactness. This reduction in AVPV is consistent with the observed decrease in apparent porosity and water absorption, indicating a refinement of the pore structure and enhanced impermeability of the GPC. Lower AVPV values are directly correlated with improved durability, as fewer permeable voids limit fluid transport and chemical attack, thereby extending the service life of the material^72^. The water absorption test, evaluated by mass gain after immersion for 30 min and 24 h, further supports these results. After immersion, the 0 wt.% TiO_2_ sample exhibited the highest mass gain, 5.42% after 30 min and 8.43% after 24 h, reflecting their higher porosity. With increasing TiO_2_ content, water uptake steadily decreased, reaching only 2.12% after 30 min and 5.34% after 24 h at 50 wt.% TiO_2_. This reduction in water absorption is consistent with the composite structure’s decreased porosity and improved packing. The localized drop in bulk density at 40 wt.% TiO_2_ (2.284 g/cm^3^) indicates a microstructural transition resulting in closed porosity. While results from ASTM C373-88 show a linear reduction in apparent porosity as TiO_2_ fills the matrix’s open pores, SEM and XRD analyses confirm that excessive filler loading at this threshold triggers significant agglomeration. Micro-scale clusters trap air pockets inaccessible to water during saturation, reducing bulk mass without affecting apparent porosity. FTIR vibrational shifts corroborate this structural distortion in the aluminosilicate framework. At 50 wt.%, the TiO_2_ crystalline phase becomes dominant, offsetting internal voids and restoring the densification trend to 2.855 g/cm^3^.

### Compressive strength

**Fig. 6:**
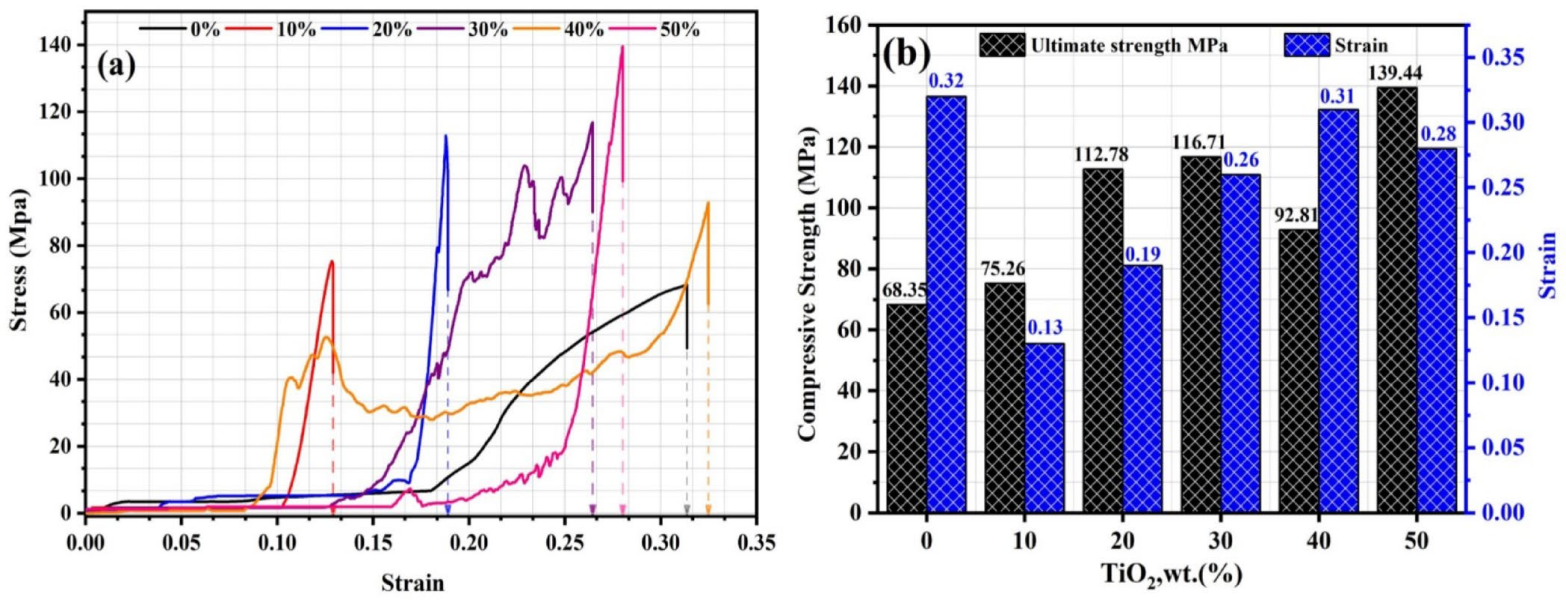
Mechanical performance of TiO_2_-modified geopolymers: (**a**) Stress–strain profiles; (**b**) Histograms compare ultimate compressive strength (UCS) and failure strain.

Compressive strength tests assessed the deformation behavior of GPC samples reinforced with 0 to 50 wt.% TiO_2_. The mechanical performance and deformation stages for each are characterized by the stress–strain profiles in Fig. 6a and the comparative histograms in Fig. 6b. The results reveal a profound structural dependency on the TiO_2_​ micro-filler concentration. The characteristic “toe region” in the strain–stress curves, most prominent in the 0% and 40% TiO_2_​ samples, reflects the initial compaction of the porous structure, which aligns with the high apparent porosity values of 33.47% and 23.7% reported for these samples. This densification, further evidenced by the increase in bulk density from 1.81 g/cm^3^ to a maximum of 2.855 g/cm^3^ upon 50% TiO_2_ addition, is a direct result of the filler effect of crystalline Anatase TiO_2_ particles, which occupy the interstitial voids within the amorphous geopolymeric framework, as confirmed by XRD patterns^73^. Furthermore, the smaller size of TiO_2_ particles, acting as nucleation points, enhances the formation of reaction products^74^. During the stable crack propagation stage, the significant shift in the Si–O–T (T = Al, Si) stretching bands observed in FTIR spectra indicates that TiO_2_ promotes a more extensively cross-linked N-A-S–H gel matrix^75^. This chemical reinforcement enhances the crack initiation threshold, allowing the 50% TiO_2_ sample to achieve a peak compressive strength of 139.44 MPa. SEM micrographs support the observed mechanical transitions, revealing a change from a porous, micro-cracked surface (0% sample) to a consolidated, integrated matrix (50% TiO_2_ composite). The anomalous behavior of the 40% TiO_2_​ sample, characterized by a lower peak stress of 92.81 MPa but a significantly higher strain of 0.31, is attributed to the localized TiO_2_​ agglomerates identified in SEM analysis. These clusters disrupt the N-A-S–H gel’s continuity, causing a localized density drop to 2.284 g/cm3. Under compression loading, agglomerated zones act as sacrificial sites, undergoing progressive crushing during unstable propagation. This explains the pseudo-ductile response and sustained residual stress observed post-peak. This synergy between the crystalline anatase phase and the dense geopolymeric binder effectively delays the final failure stage, establishing a clear structure–property correlation.

**Fig. 7:**
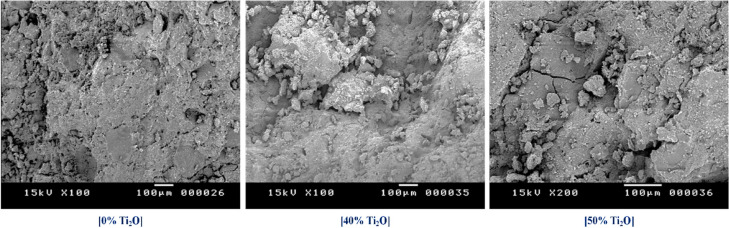
SEM images of geopolymer surfaces after compression strength tests.

Figure 7 displays SEM images of geopolymer samples containing 0, 40, and 50 wt.% TiO_2_ after performing the compressive test at the same magnification. The mechanical integrity and failure modes of kaolin-based geopolymers are significantly affected by the presence of TiO_2_ micro-fillers. The control sample 0% TiO_2_ SEM micrograph shows a fragmented morphology with extensive micro-cracks and large macro-pores. This porous structure directly correlates with the highest recorded apparent porosity of 33.47% and a relatively low compressive strength of 68.35 MPa. The absence of reinforcement allows for rapid crack propagation, as evidenced by the sharp stress drop in the corresponding stress–strain curve. For the 40% TiO_2_ sample, the micrograph reveals a distinct crushed morphology with visible clusters of microparticles. Localized TiO_2_​ agglomerates, which remained poorly integrated within the geopolymeric network, acted as stress concentration sites that underwent progressive deformation rather than discrete brittle fracture. In contrast, the 50% TiO_2_ sample retains a dense matrix even after failure. The well-embedded TiO_2_ microparticles within the aluminosilicate binder act as a rigid skeleton, resisting crack propagation and maintaining the material’s structural integrity under maximum load. According to Duan et al.^44^, mineral fillers effectively arrest micro-crack growth by forcing the cracks to deflect around the rigid particles, which explains the superior consolidation seen in the 50% sample compared to the 0% sample. The localized crushing identified in the 40% sample is a documented phenomenon in composite science. Essawy et al.^74^, noted that excessive microfiller content, if poorly dispersed, forms clusters that act as failure points, causing the crumbled appearance seen in SEM images.

### Thermal analysis

**Fig. 8:**
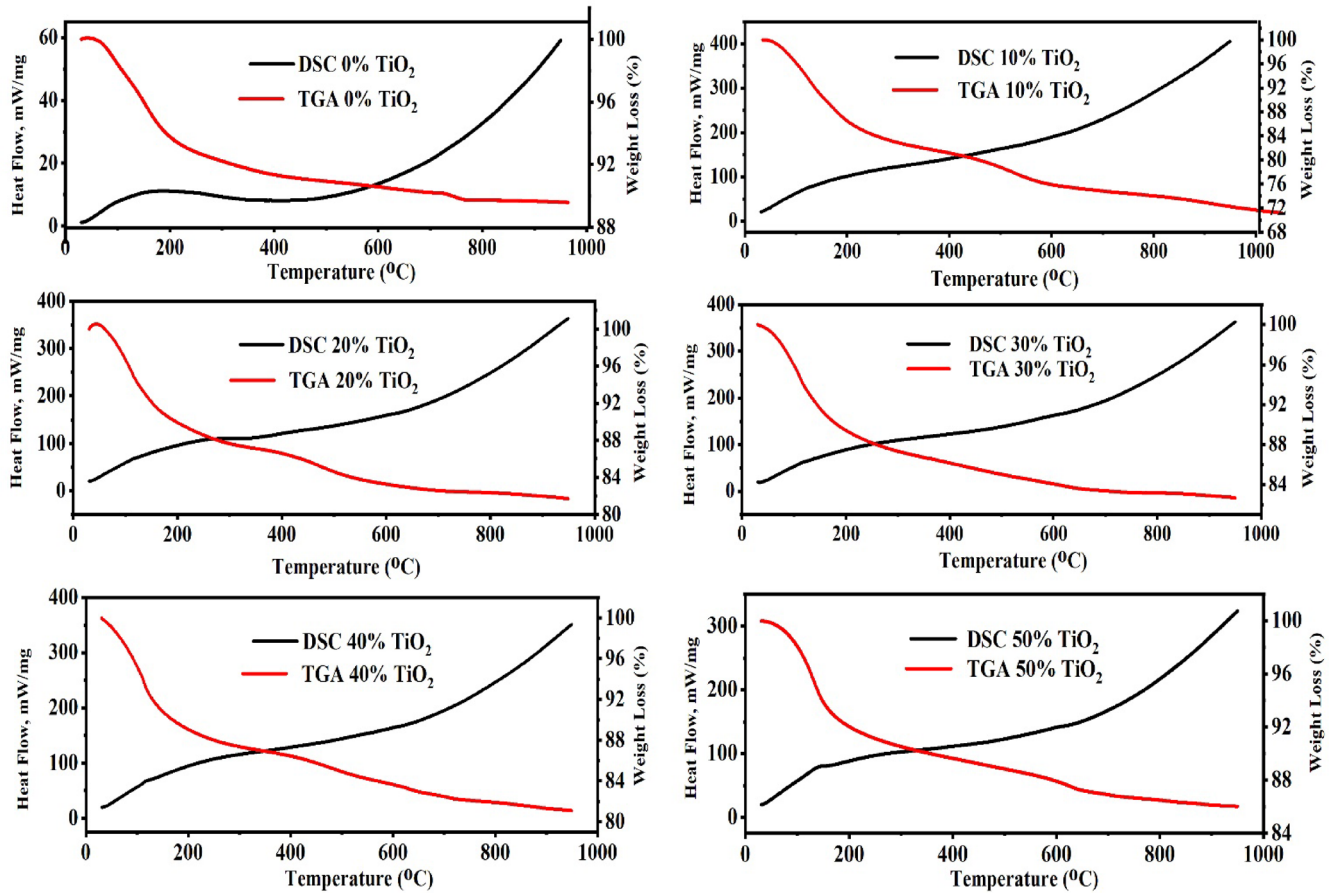
DSC and TGA profiles of GPC samples with varying TiO_2_ contents.

Figure 8 shows TGA and DSC profiles for metakaolin-based GPC containing 0–50 wt.% TiO_2_. One can show from the figure that several consistent thermochemical features are evident across the series and vary systematically with TiO_2_ loading. All samples exhibit an initial, relatively steep weight loss at low temperatures, accompanied by an endothermic DSC signal. This behavior is attributed to the desorption of physically adsorbed and capillary-held water from the geopolymeric gel and its pore network^76^. The TiO_2_-bearing samples exhibit a markedly lower mass loss in the low-temperature region compared to the control, reflecting a reduced free-water content and diminished accessible porosity, in agreement with observations from SEM and porosimetry. These phenomena indicate that TiO_2_ incorporation effectively refines the capillary pore structure, thereby limiting free-water retention within the geopolymeric matrix^77,78^.

In the intermediate temperature range from 200 to 600°C, the samples exhibit a more gradual mass loss accompanied by broad endothermic and exothermic features. The changes primarily involve dehydroxylation of bound water and condensation within the geopolymeric framework, including loss of structural –OH groups and further polycondensation of N–A–S–H species, potentially alongside removal of loosely bound organics or carbonate decomposition. Higher TiO_2_ content correlates with reduced mass loss and attenuated DSC responses, indicating enhanced thermal stability due to dilution of the reactive aluminosilicate phase by inert TiO_2_ and improved microstructural packing that restricts volatile transport^79,80^.

Between 600–1000 °C, DSC baselines typically exhibit endothermic drift, while TGA shows minimal additional mass loss. This stage may involve slow structural changes (densification, viscous flow), hydroxyl loss, recrystallization or phase separation, and the thermal stability limit of the amorphous gel. TiO_2_-enriched specimens exhibit a higher residual mass at 1000°C, evidenced by increased final weight percentage in TGA, due to the thermally stable anatase/rutile TiO_2_ fraction and reduced volatile content^57,81^.

TGA/DSC results indicate that adding anatase TiO_2_ decreases free-water content and accessible pore volume (reducing low-temperature mass loss), dilutes the reactive geopolymer gel (reducing dehydroxylation/condensation mass loss), and increases high-temperature residue due to titania’s refractory nature. These thermal trends align with XRD data (increased anatase peak intensity, diminished amorphous region) and SEM images (pore filling, densification at low-moderate loadings, and filler-dominated microstructure at high loadings)^57,78^.

### Friction studies

**Fig. 9:**
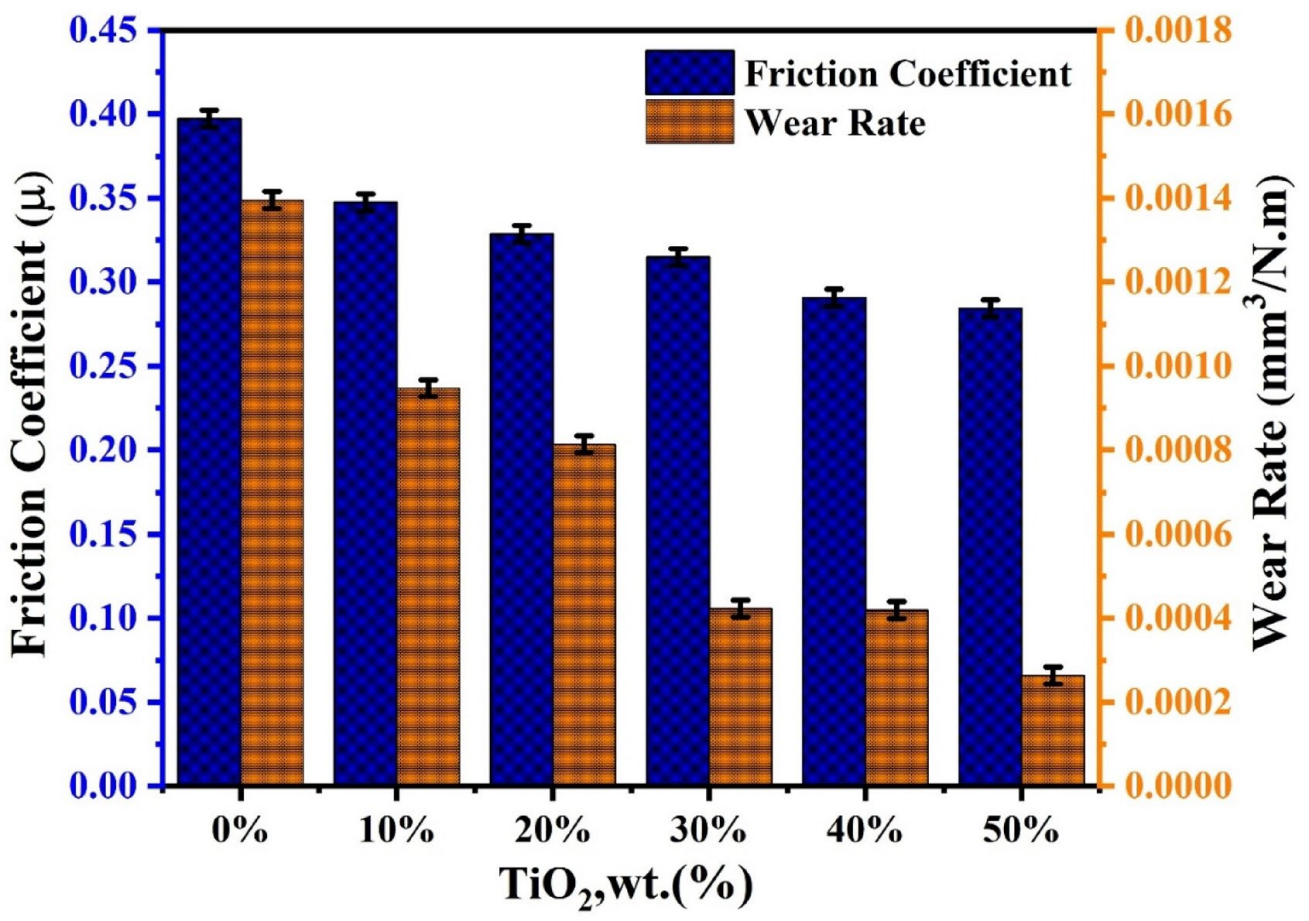
Friction coefficient and wear rate of the GPC samples with varying TiO_2_ contents.

Figure 9 exhibits the tribological performance of TiO_2_-modified GPC, as evaluated by pin-on-disc testing. The friction coefficient (µ) and wear rate both systematically decrease with increasing TiO_2_ content, demonstrating TiO_2_ effectiveness as a reinforcing filler. The tribological behavior of TiO_2_ modified GPC was systematically evaluated, revealing a strong correlation between filler loading and surface integrity. The control sample (0 wt% TiO_2_) showed the highest wear rate (1.395 × 10⁻^3^ mm^3^/N·m) and friction coefficient (0.397). The relatively high wear is attributed to the presence of unreacted metakaolin (UMK) and quartz phases identified in XRD, which create a discontinuous matrix^82^. Additionally, this behavior can be attributed to the FTIR spectra of this sample (Fig. 5), which display a prominent band at around 1019 cm^−1^, corresponding to the asymmetric stretching of Si − O − T (T = Si, Al) in the geopolymer network. Adding 10–30 wt% TiO2 significantly stabilizes the friction coefficient. This improvement correlates with the FTIR shifts observed in the 800–1100 cm^−1^ region, indicating a more cross-linked N − A − S − H gel matrix. Increasing the TiO_2_ content to 50 wt% significantly reduces the wear rate to 2.63 × 10⁻^4^ mm^3^/N·m, while the friction coefficient stabilizes at 0.284. The improvement is mainly due to pore filling and densification by TiO_2_ particles, which occupy microvoids and strengthen bonding, enhancing load-bearing capacity and hindering crack initiation. Additionally, the TiO_2_​ micro-particles act as structural fillers that densify the matrix, effectively pinning the geopolymer chains and reducing material removal during sliding contact^71^ .

**Fig. 10:**
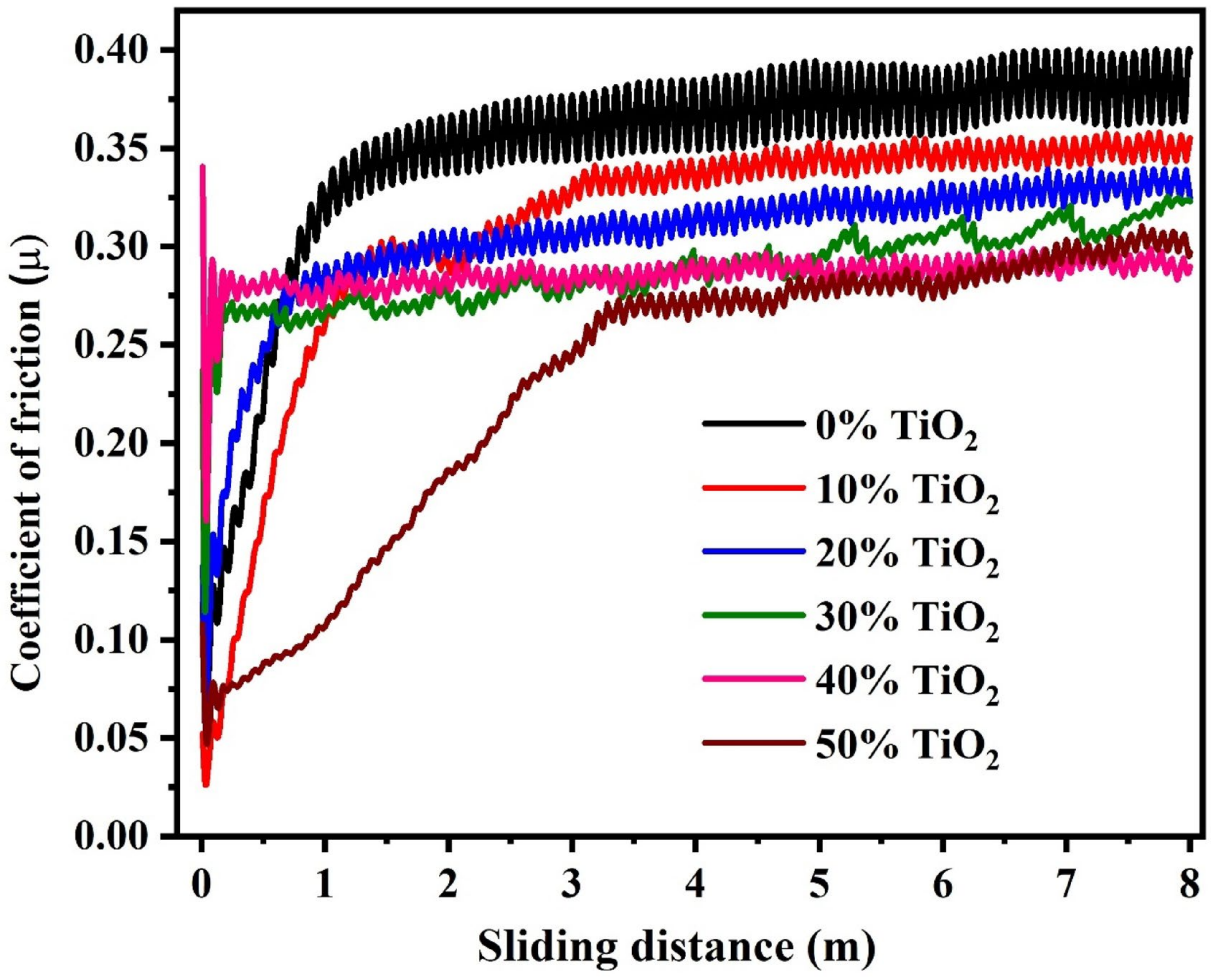
The friction coefficient versus the sliding distance curves (m) of the geopolymer composites with varying TiO_2_ additions (0%–50%).

Figure 10 shows how the coefficient of friction (µ) changes with sliding distance for GPC with varying TiO_2_ content, illustrating the impact of TiO_2_ on the material’s tribological behavior. At the beginning of the test (0–1 m), all samples exhibit a sharp increase in μ. Chemically, this represents the transition from a static state to the rupture of the initial oxide layers on the pin and disc. In the 0% TiO_2_ sample, the steeper rise to higher values (0.36–0.38) is attributed to unreacted metakaolin (UMK) and quartz particles acting as abrasive sites. In TiO_2_-modified samples, a rapid initial stabilization occurs, particularly at 20–30 wt.%, indicating that TiO_2_ particles promote the swift establishment of steady-state contact. Furthermore, the observed improvement can be attributed to the densification and pore-filling action of TiO_2_ particles, which occupy microvoids and reinforce interparticle bonding, thereby enhancing load-bearing capacity and suppressing crack initiation, as substantiated by both microstructural and durability studies^83,84^. With 50 wt.% TiO_2_, the friction coefficient initially spikes before stabilizing around 0.30. This attribute of excess TiO_2_ may dilute the reactive aluminosilicate, weakening interfacial bonding despite. The observed oscillations in the friction curves, characterized by rhythmic "ups and downs," are primarily indicative of the stick–slip phenomenon and dynamic debris entrapment at the sliding interface. Significantly high-amplitude fluctuations in the 0 wt.% TiO_2_ sample are linked to the severe brittle fracturing of the unmodified geopolymer matrix, which leads to the continuous generation of large, abrasive wear debris. In the case of the 50 wt.% TiO_2_ loading, these oscillations reappear due to the presence of extensive TiO_2_ agglomeration (AG) and micro-cracks (MC), where clusters intermittently break and reform under shear stress, causing unstable frictional resistance. Conversely, the friction curves for the 20–30 wt.% loading remain remarkably smoother and more stable, a behavior that is chemically justified by the optimal concentration of the N-A-S–H gel matrix. This dense geopolymer binder effectively anchors the TiO_2_ micro-particles, allowing them to function as stable micro-bearings that facilitate a continuous rolling effect rather than plowing, thereby minimizing vibrational noise and stabilizing the friction signal throughout the sliding distance. Coefficient of friction stabilization beyond 4 m indicates third-body tribolayer formation. FTIR and XRD analyses indicate a composite tribolayer of compacted TiO_2_ and geopolymer gel, observed by Si–O-T band shifts and anatase peaks, respectively. The highest loading at 50% TiO_2_ dip indicates initial lubrication by excess TiO_2_, succeeded by matrix fatigue due to dilution weakening filler-binder bonds. In addition, TiO_2_ improves frictional stability and lowers the steady-state coefficient, demonstrating its dual function as a structural densifier and third-body protector^85^.

**Fig. 11:**
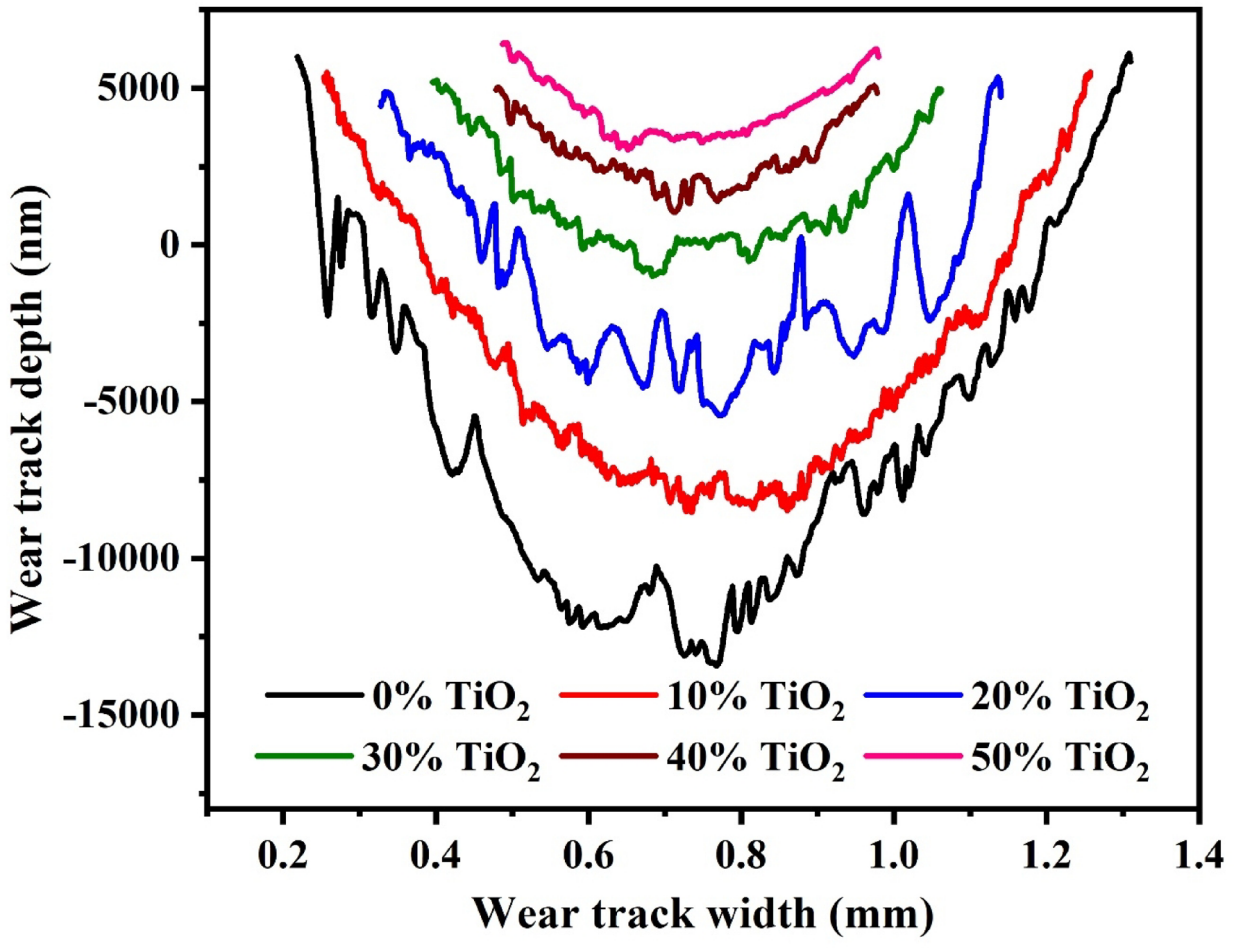
The sectional morphologies of the wear tracks of the geopolymer composites with varying TiO_2_ additions (0%–50%).

Figure 11 displays the wear track sectional morphologies of the wear pathways of the GPC at different TiO2 contents, respectively. The wear track sectional morphologies show a noticeable decrease in both depth and width as TiO_2_ is progressively added. The pristine sample (0%) exhibited high friction and erratic oscillations due to a discontinuous network of unreacted metakaolin (UMK) and quartz, resulting in severe brittle fractures. Optimal loading (20–30 wt.%) enhances cross-linking, as confirmed by Si–O–T stretching band shifts in FTIR, rigidly anchoring TiO2 micro-bearings and ensuring a smooth steady-state regime from the "running-in" stage. However, at 50 wt.%, instabilities reappear due to a dilution effect. Excessive agglomerates and micro-cracks disrupt the binder’s continuity, leading to friction signal fluctuations as the interface intermittently encounters these phase discontinuities, despite the anatase phase’s high hardness.

**Fig. 12:**
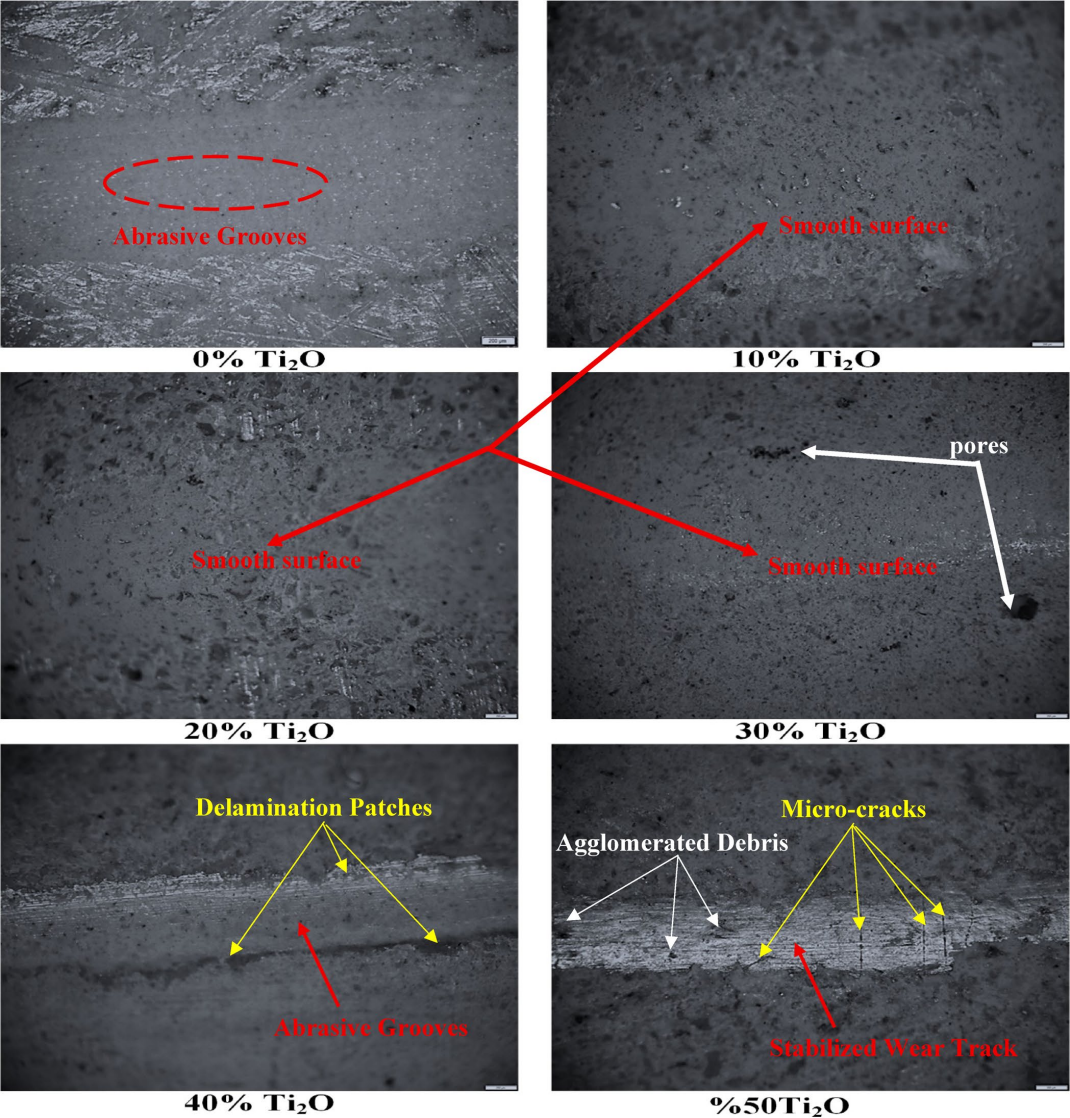
Optical micrographs of wear tracks of the geopolymer composites with varying TiO_2_ additions (0%–50%).

Figure 12 shows the optical micrographs of the worn surfaces, which corroborate the profilometry results. The unmodified geopolymer surface of the 0% TiO_2_ is characterized by prominent abrasive grooves and debris accumulation, signifying a dominant brittle-fracture wear mode. This aligns with the unreinforced nature of the pure N-A-S–H matrix. With TiO_2_ addition, the grooves become shallower and debris adhesion decreases, with the 20–30 wt.% TiO_2_ composites showing the smoothest tracks and minimal material pull-out. At higher loadings 40–50 wt.%, the surface morphology reveals a stabilized wear track at the contact zone, flanked by distinctive microcracks and agglomerated debris. The presence of these bright, sharp micro-cracks is indicative of localized embrittlement, a direct consequence of TiO_2_ agglomeration, which creates stress concentration sites within the geopolymer framework. This structural discontinuity explains the slight increase in surface roughness and friction fluctuations observed at excessive filler concentrations despite the overall high hardness. The observed transition from severe abrasive wear to a more stable mild wear regime highlights the dual role of TiO_2_ as both a microstructural densifier and a tribological stabilizer^86^.

## Conclusions

This study demonstrated that incorporating TiO_2_ into metakaolin-based geopolymers substantially improves their structural, thermal, and tribological performance. The key findings can be summarized as follows:

- Bulk density increased by 58% (from 1.81 to 2.86 g/cm^3^), while apparent porosity and water absorption decreased by 30% and 37%, respectively, indicating effective pore filling and matrix densification.
- XRD and FTIR analyses confirmed the coexistence of amorphous aluminosilicate gel with retained anatase TiO_2_, with evidence of Ti–O–Si linkages that integrate TiO_2_ into the network without disrupting the geopolymeric framework.
- SEM images revealed enhanced compactness and reduced pore connectivity at higher TiO_2_ loadings, supporting the densification mechanism.
- DSC/TGA results showed lower low-temperature mass loss and improved resistance to dehydroxylation and structural decomposition in the 200–600 °C range, confirming the stabilizing role of TiO_2_.
- Wear rate decreased significantly by 67% (from 3.45 × 10^−5^ to 1.12 × 10^−5^ mm^3^/N·m), and the friction coefficient stabilized at 0.29 compared to ~ 0.36 in the control, highlighting the dual role of TiO_2_ as both a structural densifier and a third-body tribological protector.

In conclusion, TiO_2_ emerges as a multifunctional additive that enhances the durability, thermal stability, and wear resistance of geopolymer composites, making them strong candidates for advanced applications in civil infrastructure, aerospace, and energy systems.

## Data Availability

Data supporting the findings of this study are available from the corresponding author upon reasonable request.
